# Accurate Detection of HPV Integration Sites in Cervical Cancer Samples Using the Nanopore MinION Sequencer Without Error Correction

**DOI:** 10.3389/fgene.2020.00660

**Published:** 2020-06-26

**Authors:** Wenjuan Yang, Ying Liu, Ruyi Dong, Jia Liu, Jidong Lang, Jialiang Yang, Weiwei Wang, Jingjing Li, Bo Meng, Geng Tian

**Affiliations:** ^1^Geneis (Beijing) Co., Ltd., Beijing, China; ^2^Laboratory of Genetics, Key Laboratory of Carcinogenesis and Translational Research, Ministry of Education, Peking University Cancer Hospital and Institute, Beijing, China; ^3^The Precision Medicine Centre of Drum Tower Hospital, Medical School of Nanjing University, Nanjing, China; ^4^School of Computer Science, Hunan University of Technology, Zhuzhou, China

**Keywords:** HPV, nanopore, integration, cervical cancer, next generation (deep) sequencing (NGS), third generation sequencing

## Abstract

During the carcinogenesis of cervical cancer, the DNA of human papillomavirus (HPV) is frequently integrated into the human genome, which might be a biomarker for the early diagnosis of cervical cancer. Although the detection sensitivity of virus infection status increased significantly through the Illumina sequencing platform, there were still disadvantages remain for further improvement, including the detection accuracy and the complex integrated genome structure identification, etc. Nanopore sequencing has been proven to be a fast yet accurate technique of detecting pathogens in clinical samples with significant longer sequencing length. However, the identification of virus integration sites, especially HPV integration sites was seldom carried out by using nanopore platform. In this study, we evaluated the feasibility of identifying HPV integration sites by nanopore sequencer. Specifically, we re-sequenced the integration sites of a previously published sample by both nanopore and Illumina sequencing. After analyzing the results, three points of conclusions were drawn: first, 13 out of 19 previously published integration sites were found from all three datasets (i.e., nanopore, Illumina, and the published data), indicating a high overlap rate and comparability among the three platforms; second, our pipeline of nanopore and Illumina data identified 66 unique integration sites compared with previous published paper with 13 of them being verified by Sanger sequencing, indicating the higher integration sites detection sensitivity of our results compared with published data; third, we established a pipeline which could be used in HPV integration site detection by nanopore sequencing data without doing error correction analysis. In summary, a new nanopore data analysis method was tested and proved to be reliable in integration sites detection compared with methods of existing Illumina data analysis pipeline with less sequencing data required. It provides a solid evidence and tool to support the potential application of nanopore in virus status identification.

## Introduction

Human papilloma virus (HPV) is the major cause of cervical cancer, and HPV16 and 18 are the two most prevalent high-risk HPV types worldwide. Cervical cancer is the second most common malignant cancer in females; 570,000 women are diagnosed with cervical cancer and 311,000 die of this disease each year (Bray et al., 2018). Although it is known that persistent HPV infection causes cervical cancer, it remains unclear how HPV induces carcinogenesis and what exactly plays the most important function in the process. After HPV infection, HPV proteins E6 and E7 are expressed, which inhibits tumor protein 53 and retinoblastoma protein, disrupts cell proliferation and many other biological processes, and induces cervical cancer (Balasubramaniam et al., 2019). In addition, HPV DNA could also integrate into human DNA, which is an early and important event during carcinogenesis (Nicolas et al., 2004; Pett and Coleman, 2010; Schmitz et al., 2012; Christiansen et al., 2015; Bodelon et al., 2016; Walline et al., 2017), and involves epigenetic mechanisms that affect the expression of key genes in the tumor transformation process. Maria et al. (2015) proposed a scheme of modifications and alterations generated by HPV integration into the host genome that can lead to carcinogenesis. This recent evidence shows that HPV integration often preferably affects those genes that are continuously expressed during DNA transcription and repair to induce carcinogenesis (Oyervides-Muñoz et al., 2018). In addition, the progression to cancer can be explained by viral DNA integration into tumor suppressor genes; this integration into host DNA inactivates those genes leading to uncontrolled growth (Zhao et al., 2016). Understanding viral oncogenesis is critical for the clinical management of HPV-positive cancer (Morgan et al., 2017).

An increasing number of studies have indicated that detecting HPV DNA integration has become mainstream in HPV oncogenic research worldwide, leading to the discovery of HPV integration into the human genome (Svetlana et al., 2008; Hu et al., 2015; Zhang et al., 2016; Shen et al., 2017; Ibragimova et al., 2018; Warburton et al., 2018). Hu et al. (2015) found that HPV randomly integrates into the human genome with the tendency to integrate into genomic hot spots. Svetlana et al. (2008) found that HPV integration sites significantly vary among HPV types. Warburton et al. (2018) found that HPV integration generates a super-enhancer-like element composed of tandem interspersed copies of the viral upstream regulatory region and a cellular enhancer, which drive high levels of oncogene expression. Zhang et al. (2016) and Shen et al. (2017) discovered that HPV integrated into the human genome can significantly increase related gene expression. Ibragimova et al. (2018) and Koneva et al. (2018) discovered that patients with integrated HPV status have a worse survival rate compared to those with episomal HPV. The increasing number of studies about HPV integration has stimulated the idea that HPV integration status may be a biomarker for the diagnosis, progression, and survival prediction, and even as a biomarker for cancer screening (Han et al., 2015; Liu et al., 2018; Grayson et al., 2019; Jiang et al., 2019). Harlé et al. (2019) found the same integration sites and DNA deletion regions between tongue and anal cancer of the same patient. Campitelli et al. (2012) analyzed the serum signal of HPV integration sites and found that it could be used as a liquid biopsy marker to monitor the effects of individual treatment plans. HPV integration status and locus discovery have become increasingly important not only for discovering the mechanism underlying virus infection, but also for application in clinical diagnosis and treatment to decrease the incidence and improve cervical cancer treatment.

Different methods have been used to study HPV integration sites including amplification of papillomavirus oncogene transcripts (APOT), detection of integrated papillomavirus sequences by ligation-mediated PCR (DIPS-PCR), and next-generation sequencing (NGS). Das et al. (2012) found 48 integration sites by APOT, which is a method based on RNA level detection of HPV integration; therefore, the results greatly rely on the sample quality. Luft et al. (2001) discovered 22 integration sites by DIPS-PCR including known and new integration sites. Although it is a method based on DNA detection, the potential for identifying new integration sites is limited. With the development of NGS, whole genome sequencing has been used for virus integration sites detection (Tuna and Amos, 2016; Chae et al., 2018). However, it requires large amounts of sequencing data, and thus is not applicable in clinical usage, which requires fast and accurate results. To date, the best way to detect integration sites is the probe capture sequencing method. After enrichment of virus genomic material using this method, the fusion fragment of human and HPV sequence is isolated and further sequenced by NGS. Many studies have used this method to identify large amount of integration sites with good accuracy (Chandrani et al., 2015; Liu et al., 2016a, b; Li et al., 2018, 2019). Although NGS is powerful for discovering HPV integration sites, the quality of the results relies on many factors including the probe capture efficiency, sequencing depth, and analysis method. In addition, the requirement for rapid and accurate results for clinical application is not met with this method. Therefore, the development of a new method for HPV integration detection with high accuracy and prompt reporting capacity is crucial for future clinical application.

In this study, we developed a novel method to detect HPV integration sites using the third generation nanopore sequencing platform. We compared our detection results with previously published and newly generated Illumina results. Our results showed that third-generation sequencing technology can be used to detect HPV integration sites.

## Materials and Methods

### Sample Collection

A fresh tissue specimen was collected from a patient with IIIb squamous cell carcinoma who had undergone surgery at Anyang Cancer Hospital (Henan province, China) in 2009. The patient was 56 years old and had HPV16-positive cancer. This study received ethical approval from the Institutional Review Board of the hospital. Individual informed consent had been collected from this study participant.

### DNA Preparation

Genomic DNA, provided by Peking University Cancer Hospital and Institute (Beijing, China), was extracted using the DNeasy Blood & Tissue Kit (Qiagen, Hilden, Germany) according to the manufacturer’s protocol. Double-stranded (ds) DNA was quantified using the Nanodrop 2000 and Qubit dsDNA HS Assay Kit (both from Thermo Fisher Scientific, Inc., Waltham, MA, United States). The average fragment size of DNA [>5 kilobase pair (kbp)] was measured (identified by comparison to DL2000 PLUS DNA Ladder, Life Technologies, Carlsbad, CA, United States) on a 1.0% agarose gel in 1X TAE buffer on the Bio-Rad CHEF DRII system.

### Illumina Library Preparation

Sheared DNA was used to make genomic DNA libraries using the NEBNext^®^ UltraII^TM^ DNA Library Prep Kit for Illumina^®^ (E7370L) and NEBNext^®^ Multiplex Oligos for Illumina^®^ [E6609L; New England Biolabs (NEB), Ipswich, MA, United States] as recommended by the manufacturer. In short, 200 ng DNA was sheared in a 50 μL volume using the Covaris M220 Focused-ultrasonicator (Covaris, Cambridge, MA, United States) for 340 s with a duty cycle of 10%, cycles per burst of 200, and peak power of 75. Then, fragmented DNA was end-repaired and A-tailed using the NEBNext^®^ UltraII^TM^ Mix and buffer, and ligation of DNA adapters was done using the NEBNext^®^ UltraII^TM^ Ligation Master Mix and Enhancer. Then, 0.9 × AMPure XP beads (Beckman Coulter Inc., Skyesville, MD, United States) were used to remove all traces of adapter dimers. The adapter-ligated DNA was amplified with six cycles of PCR using the NEBNext^®^ UltraII^TM^ Q5 Master Mix. The generated library was quantified with the Qubit dsDNA HS Assay Kit (Thermo Fisher Scientific) using the Qubit 3.0 fluorometer (Invitrogen, Carlsbad, CA, United States), and run on the Qsep1^TM^ biosystem (BiOptic Inc., NTC, Taiwan) for quality analysis. The final size of the electrophoresis fragment was about 320 bp.

### Targeted Capture and Illumina Sequencing

Human papillomavirus probes were designed by Integrated DNA Technologies (Integrated DNA Technologies, Coralville, LA, United States), according to the full-length genome of HPV16. Overall NGS target enrichment with xGen Hybridization and a Wash Kit was conducted by Integrated DNA Technologies, which is briefly described below and performed as detailed by the plate standard protocol for xGen^®^ hybridization capture of DNA libraries from Integrated DNA Technologies. In brief, whole-genomic libraries were hybridized with HPV probes (Integrated DNA Technologies), absorbed onto the beads via Dynabeads MyOne Streptavidin T1 (Thermo Fisher Scientific), after which the uncaptured DNA fragments were removed by washing. Then, the eluted fragments containing the targeted gene were enriched by 15 cycles of PCR to generate libraries for sequencing. The captured library was quantified with the Qubit dsDNA HS Assay Kit using a Qubit 3.0 fluorometer (both from Thermo Fisher Scientific) and run on the Agilent 2100 TapeStation (Agilent Technologies, Santa Clara, CA, United States) for quality analysis prior to sequencing. DNA libraries were sequenced using the NextSeq platform (Illumina) with 150 bp paired-end reads.

### Library Preparation for Nanopore Sequencing

The sheared DNA was used to make genomic DNA libraries using the NEBNext^®^ UltraII^TM^ DNA Library Prep kit for Illumina^®^ (E7370L) and NEBNext^®^ Multiplex Oligos for Illumina^®^ (E6609L), as recommended by the manufacturer, followed by DNA shearing with the M220 Focused-ultrasonicator (Covaris). Then, 200 ng genomic DNA was sheared into longer fragment sizes of about 800 bp in a 50 μL volume on the Covaris M220 (Covaris) for 50 s with a duty cycle of 20%, cycles per burst of 200, and peak power of 50 according to the manufacturer’s instructions. Then, the fragmented DNA was end-repaired and A-tailed using the NEBNext^®^ UltraII^TM^ Mix and buffer, and ligation of adapters was done using the NEBNext^®^ UltraII^TM^ Ligation Master Mix and Enhancer, after which 0.9X AMPure XP beads (Beckman Coulter) were used to remove all traces of adapter dimers. The adapter-ligated DNA was amplified with six cycles of PCR using the NEBNext^®^ UltraII^TM^ Q5 Master Mix. The generated library was quantified with the Qubit dsDNA HS Assay Kit using the Qubit 3.0 fluorometer (both from Thermo Fisher Scientific) and run on the Qsep1^TM^ biosystem (BiOptic) for quality analysis. The final size of the electrophoresis fragment ranged from 250 to 1500 bp. The overall capture experiment with xGen Hybridization and Wash Kit was conducted by Integrated DNA Technologies as detailed by the standard protocol for xGen^®^ hybridization capture of DNA libraries from Integrated DNA Technologies with slight modifications. Briefly, the HYB program extends for 4 to 6 h that incubating the tubes at 65°C with a heated lid set at 105°C. The captured library was quantified with the Qubit dsDNA HS Assay Kit using the Qubit 3.0 fluorometer (both from Thermo Fisher Scientific) and run on the Agilent 2100 Tape Station (Agilent Technologies) for quality analysis prior to nanopore sequencing. DNA libraries were sequenced using MinION^TM^ MK1B device from Oxford Nanopore Technologies (ONT; Oxford, United Kingdom).

### Nanopore Sequencing

The capture of HPV 16 probe-template duplexes was done using the Ligation Sequencing Kit (SQK-LSK108) with Native Barcoding Expansion (EXP-NBD103) from ONT, and performed as detailed by the 1D Native barcoding genomic DNA standard protocol from ONT. Then, 310 ng purified amplicon DNA was dA-tailed using the NEBNext Ultra II End Repair/dA Tailing module (E7546S; NEB) at a temperature of 20°C for 30 min and at 65°C for 30 min using the thermal cycle. Then the DNA was purified with AMPure XP beads (A63881; Beckman Coulter), and 60 μL (1×) of re-suspended AMPure XP beads was added by pipetting and thoroughly mixed on a rotator mixer for 5 min. The beads were separated on a magnet to remove the supernatant. The beads were kept on the magnet and washed twice using 200 μL of 70% ethanol without disturbing the pellet, followed by re-suspension of the pellet in 25 μL nuclease-free water. Then, 275 ng end-prepared DNA was ligated with native barcode NB04 from ONT using the NEB Blunt/TA Ligase Master Mix (M0367S; NEB), and then incubated at 25°C for 15 min. Following the barcode ligation reaction, the DNA was cleaned again with 1X Agencourt AMPure XP beads (Beckman Coulter) and eluted in 26 μL. The resulting DNA was pooled with another unrelated barcoded library by equivalent weight. Then, 543 ng pooled barcode DNA was used to perform the adapter ligation step, and 20 μL Barcode Adapter Mix, 10 μL Quick T4 DNA Ligase, and 20 μL of NEB Next Quick Ligation Reaction Buffer (5X; E6056S; NEB) were added in that order to the pooled barcoded 50 μL DNA. The reaction was incubated at room temperature for 15 min, and then cleaned using 0.4X Agencourt AMPure XP beads (Beckman Coulter). Then, the beads were washed twice with 140 μL Adapter Bead Binding, re-suspended in 15 μL Elution Buffer, and incubated for 10 min at room temperature before pelleting in a magnetic rack. The prepared library was combined with 35 μL running buffer with Fuel Mix, 25.5 μL Library Loading beads and 12 μL DNA library (∼158 ng) and loaded into the SpotON sample port of the R9.4 flowcell. All nanopore sequencing runs were conducted using the MinION^TM^ MK1B device following the recommended sequencing protocols by ONT. The captured library was sequenced individually using the FLO-MIN-106 flow cell. MinKNOW software (v18.07.18) was used to control the MinION device following a 24-h run script, and sequencing data were collected in real-time and processed into basecalls using the Metrichor^TM^ agent.

### Bioinformatic Analysis

The nanopore sequencing results were basecalled using the EPI2ME interface (v. 2.59.1896509). For passed 1D reads, quality scores (Q-score ≥ 7) and length distributions were evaluated using FastQC. Obtained fastq files were converted to fasta files using the FASTX-toolkit^1^. These fasta files were aligned to the Illumina adapter database by LAST (version: lastal 956; Frith et al., 2010; Kiełbasa et al., 2011), which alignment parameters were mismatch cost: 1, gap extension cost: 1, gap existence cost: 1, and minimum score for gapped alignments: 45. Then these reads were divided into two group reads, which we defined that the only library (OL) structure reads and multiple library (ML) structure reads (Figure 2B). The ML reads were split to the OL reads by the Illumina adapter sequences. All OL reads were aligned to the human genome (GRCh37/hg19) and HPV genome (NC_001526.2) databases using Blat (version: v36; Kent, 2002), with parameters were stepSize = 5, repMatch = 2,253, minScore = 20, and minIdentity = 0. We also defined the highest score alignment result as the best OL reads alignment result to find the breakpoint of HPV integration. We used Samtools (version: v1.9) software to analyze the HPV16 genome sequencing depth. In each one OL reads, we defined the breakpoint of HPV integration which the gap and overlap between the best human alignment result and the best HPV alignment result less than 10 bp. The breakpoint was annotated to gene function using ANNOVAR^2^ and an in-house program with human reference and HPV16 reference, respectively. The sequencing reads generated by Illumina sequencer were analyzed using the SEGF pipeline (Xu et al., 2018). A diagram of the integration site distribution was made using Illustrator software. Gene function analysis was applied using the DAVID online analysis tool (Huang et al., 2009a, b).

### PCR Verification

To validate the selected HPV integration into the human genome detected by nanopore sequencing, primers were designed, one of which was derived from the human genome at the potential site of integration, and the other of which was against HPV sequences suspected of being near the site of integration within the HPV genome. Both primers were designed 100∼400 bp away from the detected integration sites based on the nanopore targeted-capture reads, and 13 HPV integration sites (*n* ≥ 5) were selected. PCR was performed using each primer set for DNA that was previously used for HPV capture. As a template, 1 μL (30 ng) genomic DNA solution was used in the subsequent PCR. The PCR reaction mix was prepared in a total volume of 20 μL containing 4 μL 5X Phoenix Hot Start Taq Reaction Buffer, 2 μL dNTP (2.5 mM), 0.5 μL of each forward and reverse primer (10 mM), 0.2 μL Phoenix Hot Start Taq DNA Polymerase (500 U), and 11.8 μL nuclease-free water (not DEPC-Treated). The PCR conditions were as follows: 5 min at 95°C; 35 cycles of 30 s at 94°C; 60 s at 50–60°C for annealing; and 60 s at 72°C; followed by 72°C for 1 min. PCR products were analyzed by electrophoresis on 2.0% agarose gels, purified, and Sanger sequenced.

## Results

### Nanopore and Illumina Sequencing Results

**FIGURE 1:**
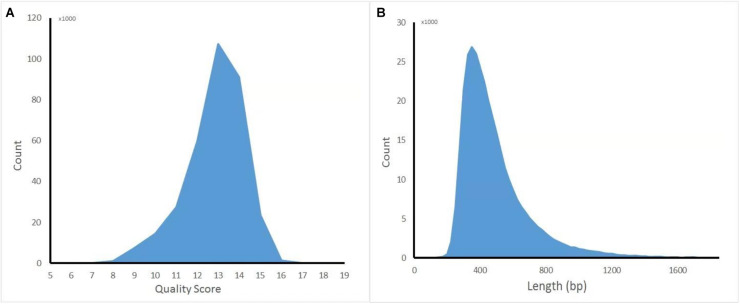
Distribution diagram of nanopore sequencing quality score and read length. **(A)** Quality score. **(B)** Length.

The captured library was constructed and sequenced by ONT MinION. The sequencing run generated 381,475 (149.1 Mb) sequencing reads from 977 active pores. To obtain high-quality reads, the raw reads were filtered using the Metrichore 1D base calling program and kept for further analysis if a Qscore ≥ 7 was obtained. In total, 333,028 1D reads (130.2 Mb) were retained with the read lengths ranging from 67 to 7,404 bp, with a mean read length of 502.8 bp, and the quality score ranged from 7 to 17, with a mean value of 12.8. The distribution diagram of the read lengths and quality scores are shown in Figure 1 and other sequence details are summarized in Table 1. The captured Illumina library was sequenced by Illumina sequencing machine and 11,093,630 raw reads (1.67 Gb raw bases) were generated. After data cleaning, 11,093,535 clean reads (1.64 Gb) were obtained with an average length of 151 bp. The summary of sequencing results from the two platforms are listed in Table 1 and the sequence coverage of HPV16 genome by Illumina and nanopore sequencer was shown in Supplementary Figure S1. It showed that HPV16 full genome was covered by both sequencing platforms and Illumina result provided obviously higher sequence depth than nanopore. The sequence depth of the most HPV16 genome regions are even both on Illumina and nanopore results, excepting a small region around 4,000 bp location in Illumina. Although it showed a significant decrease of the sequence depth in this region, it provided more than 100× sequencing depth of it.

**TABLE 1:** Summary of the sequencing results of HPV integration sites by nanopore and Illumina platforms.

Platform	Raw reads	Quality filtered reads (≥Q20)	Pass 1D reads	Length (bp)			Sequence quality Scores		
				min	mean	max	min	Mean	Max
Illumina	11,093,630	11,093,535(99.9%)	/	/	151	/	/	/	/
Nanopore	381,475	/	333,028(87.3%)	67	502.8	7,404	7	12.8	17

### Identification of HPV Integration Sites by Nanopore and Illumina Platforms

**FIGURE 2:**
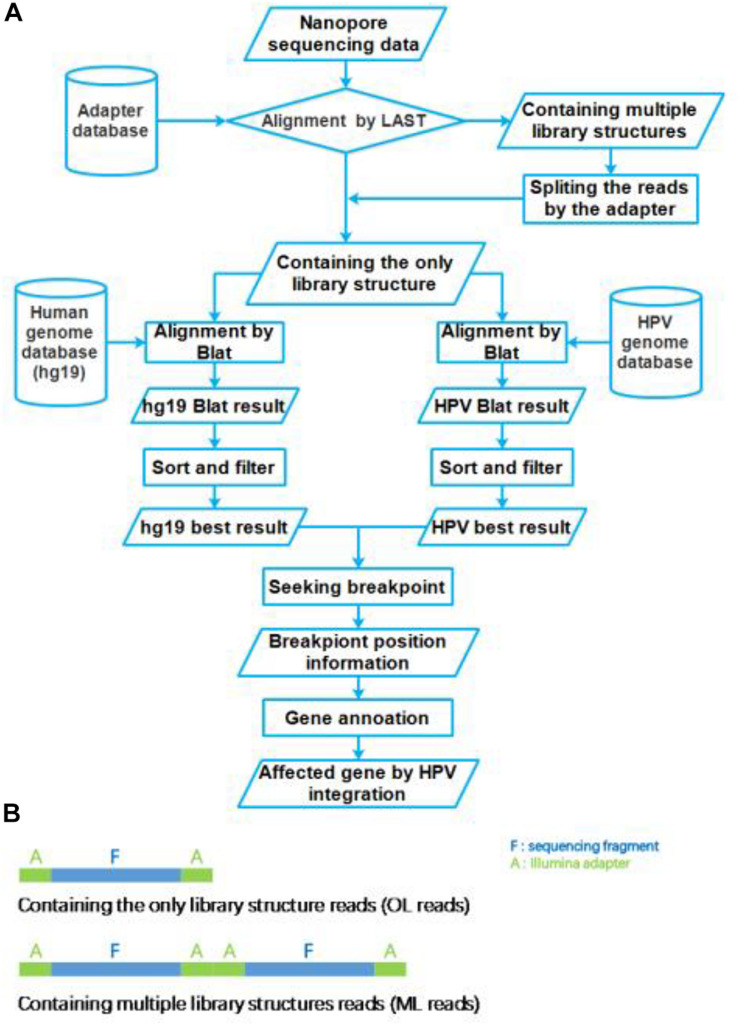
Flow chart of HPV integration site analysis pipeline. **(A)** The workflow of HPV nanopore sequencing analysis. **(B)** The library structure of nanopore sequencing reads.

**FIGURE 3:**
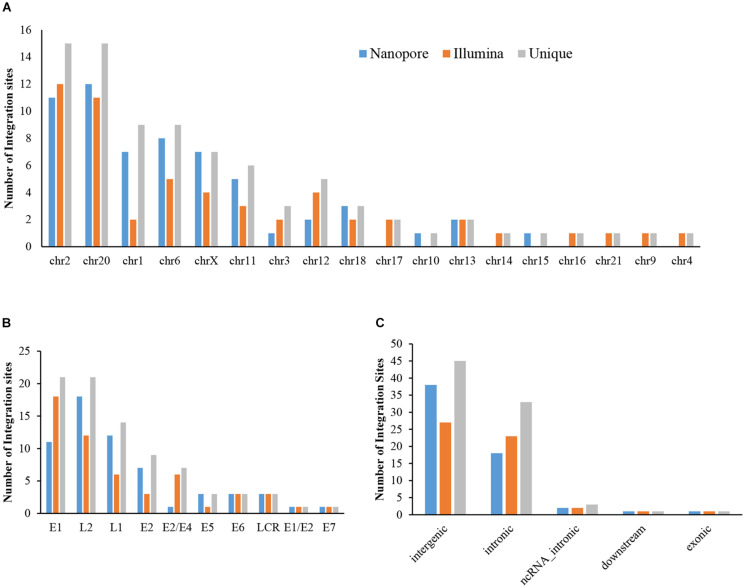
Distribution of integration sites identified by Nanopore and Illumina platforms. **(A)** HPV integration breakpoints distribution in human chromosomes. **(B)** HPV integration breakpoints distribution in the HPV genome. **(C)** HPV integration sites functional locations in the human genome.

The sequencing data from nanopore sequencing were analyzed according to the pipeline described in the Materials and Methods, shown in Figure 2A. There were 12,951 sequence reads in the human genome and 389,839 reads in the HPV genome. Next, the potential integrated reads were analyzed using BLAST with the human and HPV genome sequences to determine the exact integration breakpoints. Without performing error correction analysis, 7,859 reads were identified in both the human and HPV sequences. To filter out the chimeric product from the library construction process, we used the number of overlapped bps as filter criteria. The HPV and human part of the same sequencing read were identified with different bp-length gaps. Only reads, whose gap was shorter than 10 bp length, were kept for further analysis. A total of 339 integration sites were finally identified (Supplementary Table S1). The location and gene information of the matched sequence in either the human or HPV genome were annotated. The read number of unique breakpoints was calculated and only those breakpoints with ≥ 2 reads identified were kept for further comparison analysis with two closed sites (HPV16:3327,chr6:7328094 and HPV16:3329,chr6:7328093)combined. Excepting 2 site also identified by Illumina with 3 reads (HPV16:4405,chr2:99438640 and HPV16:2716,chr13:74250432; Supplementary Table S2). There were 60 breakpoints in total were included. The distribution of all breakpoints in the different chromosomes is shown in Figure 3A. Chromosomes chr20, chr2, chr6 were the three chromosomes with 12, 11, and 8 breakpoints, respectively. Both chr1 and chrX had 7 and 7 integration sites each and the rest of the chromosomes had ≤5 integration sites. The identified read numbers of each integration site were highly different, with a range change from 2 to 406. There were 31.7% (19 of 60) integration sites had more than 10 reads. The top 19 abundant integration sites were distributed in chromosomes 20(6), 6(4), X (4), 11(3), and 2(2). Among the top five most abundant breakpoints, There were 3 located in chromosome 20, HPV16:2804,chr20:32516985 (406 reads), HPV16:7139,chr20:32478733 (169 reads), HPV16:4276,chr20:32502143(51 reads), and two were in chrX, HPV16:5534,chrX:20464412 (132 reads), HPV16:3163,chrX: 20462930 (50 reads). Figure 3B indicates the integration site distribution in the HPV genome. Within the 60 identified integration sites, 18 were in the L2 gene, 12 in L1, 11 in E1, 7 in E2, and <4 in the each of the other genes (E6, LCR, E5, and E7). On the human genome, there are 38 integration sites in the intergenic region, 18 in the intronic region, 2 in the ncRNA region, 1 in the exonic and 1 in the downstream region of the genes, as indicated in Figure 3C. To further look at the breakpoints in the same chromosome, in chr20, most breakpoints (10 of 12) located in the intergenic region of two genes *CHMP4B*, *RALY-AS1*, one of the other two breakpoints in intergenic region of *KIF3B*, *ASXL1*, and the other is in the intronic region of gene *ATRN*. With the exception of chr20, other chromosomes also have obvious cluster tendency of integration sites, like six integration sites of chrX in the intergenic region of genes *RPS6KA3* and *CNKSR2*. Three breakpoints in chr6 were in the intronic or downstream of gene *CAGE1*. 10 regions of human genome were identified with more than two breakpoints clustered together, indicating there were integration sites cluster in the human genome. In addition, 14 breakpoints without any cluster tendency were observed. Illumina identified 1,718 integration sites, and only the integration sites with ≥ 3 reads were collected for further analysis. All sites are listed in Supplementary Table S2. The characteristic analyses of these integration sites are summarized in Figure 3.

### Integration Sites Difference of All Three Data Sets

**FIGURE 4:**
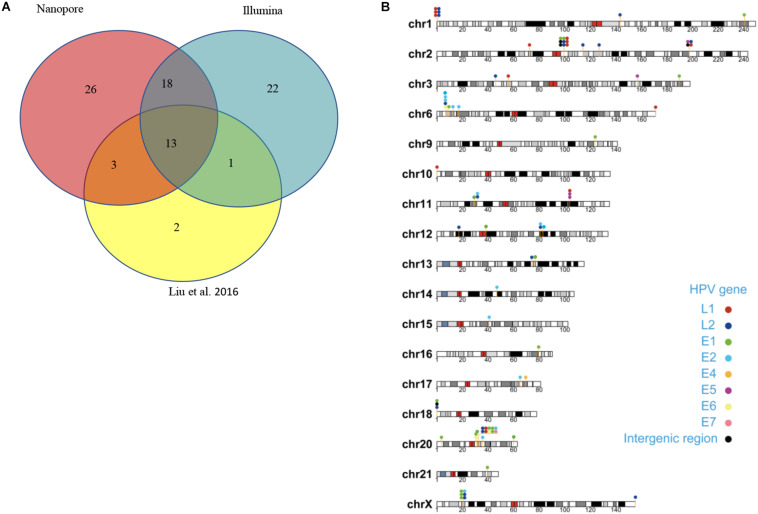
Summary of unique integration sites. **(A)** Venn diagram of overlapping integration sites of two identified methods. **(B)** Chromosome localization of unique integration sites from three datasets.

**FIGURE 5:**
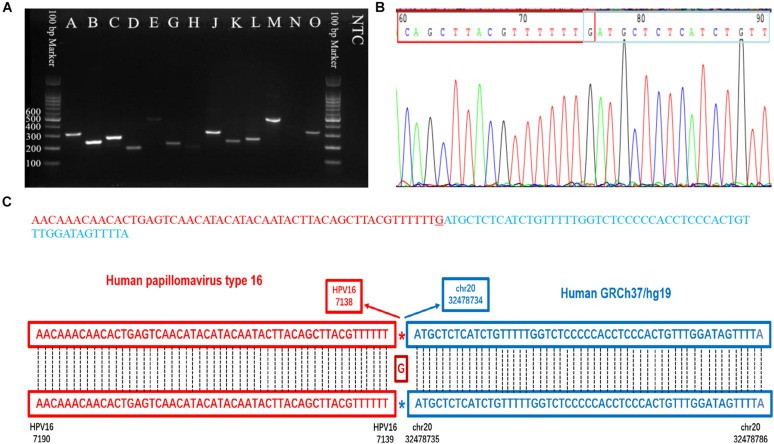
PCR gel of verified integration sites and sequencing result of integration site D. **(A)** Gel image of amplified integration sites DNA fragments. **(B)** Sanger sequencing of integration site D. **(C)** The sequence and blast result image of the integration site D.

Capture-based Illumina identified 54 integration sites with ≥ 3 reads sequenced, and four sites (HPV16:7619,chr2:99404946 and HPV16:6558,chr2:99431140, HPV16:850,chr20:32515476, and HPV16:462,chr20:30942164) with less than three but overlapping with nanopore results showed in Table 2. Altogether, there are 54 integration sites were included for further analysis. A previously published paper (Liu et al., 2016a) reported 19 integration sites from the same sample we used. All integration sites were compared and the overlapping sites by either method were recorded. The numbers are shown in the Venn diagram in Figure 4A, and the detailed list of integration sites is shown in Figure 4B and Table 2. There were 13 integration sites identified by all three platforms, which indicated good repetitiveness between the different platforms. A total of 18 sites overlapped by nanopore and Illumina, three overlapped by nanopore sequencing and in the previous paper, and only one overlapped by Illumina and the published paper. Among the identified integration sites in the two platforms, some breakpoints had highly abundant read numbers, such as HPV16:2804,chr20:32516985 with 406 reads in the nanopore results and 1439 reads in the Illumina results. Other breakpoints had very few reads numbers such as HPV16:4250,chr21:97550764 with two reads in the nanopore results, whereas there were only four reads in the Illumina results. Besides the overlapping sites, each platform had their own unique integration sites. Nanopore, Illumina, and Liu et al. had 26, 22, and 2 unique integration sites, respectively. A total of 6 of the 26 nanopore integration sites were highly abundant with ≥ 6 reads sequenced, namely HPV16:7139,chr20:32478733 (169 reads), HPV16:4276, hr20:32502143 (51 reads), HPV16:4029,chr11:103891933 (21 reads), HPV16:3312, chr6:17081322 (8 reads), and HPV16:3295,chr11:31761709 (6 reads), HPV16:5311, chr1:455824 (6 reads). Within the integration sites identified by all the three methods, Illumina, nanopore and Liu paper, with relative reliable abundance, nanopore identified the most validated integration sites. All the unique integration sites and their detailed information are listed in Table 2. To test the accuracy of the new integration sites identified by our data, we performed Sanger sequencing. Together, 14 integration sites were chosen for verification, and 13 were PCR-amplified and successfully sequenced, indicating the true positive of these integration sites, which were mostly identified by both nanopore and Illumina platforms. Detailed information on the designed primers and sequencing results with chromas images are summarized in Supplementary Table S3 and Supplementary Figure S2. The verified integration sites show different coexisting situations. Eight coexisted in nanopore and Illumina integration sites datasets, which indicating the advantage of our integration detection pipeline of both platforms compared with previouse published pipeline. There were 9 integration sites with more than 10 identified reads in each of the three datasets, and four integration sites with less than 10 reads in all three datasets were verified. The high verification rate (92.86%) of integration sites also indicated the high integration sites detection accuracy. Also, four sites were verified to exist in the intronic region, and the remaining nine integration sites were found to exist in the intergenic region. The PCR gel image and the sequencing result were shown in Figure 5 and Supplementary Table S3, respectively.

### Affected Gene Function Analysis

All the integration sites identified by the three platforms were combined to the final unique integration sites list (Table 2) for further analysis with nanopore reads (*n* ≥ 2) and Illumina reads (*n* ≥ 3). A total of 83 unique integration sites were found, each of which were annotated, and further function classification and pathway analyses were conducted. If the integration sites located in intergenic region, the closer gene was used for analysis. The genes were clustered into eight biology processes including positive regulation of transcription from RNA polymerase II promoter (6 genes), negative regulation of transcription from RNA polymerase II promoter (6 genes), regulation of transcription from RNA polymerase II promoter (5 genes), transcription from RNA polymerase II promoter (4 genes), negative regulation of retinoic acid receptor signaling pathway (2 genes),establishment of skin barrier (2 genes), proximal/distal pattern formation (2 genes) and embryonic limb morphogenesis (2 genes) and six cytoplasm (13 genes), nucleoplasm (8 genes), extracellular exosome (8 genes), protein binding (6 genes), DNA binding (2 genes) and actin binding (2 genes). Detailed information on the function classification results is shown in Supplementary Figure S3 and Supplementary Table S4.

## Discussion

Most studies have indicated that nanopore sequencing results contain about a 10–15% error rate (Nagarajan and Pop, 2013; Melanie et al., 2015), which greatly limits the application of the nanopore platform in the genome study, especially in clinical applications. Although some studies have indicated that error correction can significantly improve the assembly result and help with finding small variations in the genome (Lu et al., 2016; Sović et al., 2016; Xiao et al., 2017), choosing appropriate error correction software can be challenging for researchers who are not good at bioinformatics analysis. In addition, different error correction software provides different results that can potentially affect the study results. In our study, we combined two steps of different sequence matching analyses, Last and Blat, and after comparing the results with previous verified integration sites, we obtained 16 correct integration sites in 19 positive ones, strongly indicating that our data analysis method is appropriate for integration site discovery analysis by using nanopore sequencing data. Last can determine the human and virus merged sequence promptly and based on the candidate reads, and Blat can help identify the exact break point position accurately. With stringent criteria used for filtering, we filtered out the chimeric PCR product and only kept the correct structured integration sites. Therefore, the results were compatible with the positive one. However, to obtain high capture efficiency when enriching integration sites by using an HPV probe, we only used 500 bp reads for library construction. It has been reported that genome structure might be complex if two integration sites located closely in a genome region (Michael et al., 2014; Walline et al., 2016) and has been found in long sequencing fragment, e.g., 5 kbp (Cretu Stancu et al., 2017). Although we intended to discover the whole structure of virus integration in the human genome as paper reported (Adey et al., 2013; Akagi et al., 2014), the results did not support our hypothesis mainly due to the reason that the reads were not long enough to detect multiple integration sites within one region. To improve it, we need to either try longer reads library construction by HPV probe capture in the future.

**TABLE 2:** The summary of identified integration sites by all three datasets (Nanopore/Illumina/Liu et al.) after reads number filtration.

Breakpoint*	Reads number (N/I/Liu)	HPV16 gene	Chromosome	Func_refGene	Gene_refGene	GeneDetail_refGene (bp)	Map	Sanger Verified
HPV16:2804,chr20:32516985	406/1439/940	E1/E2	Chr20	Intergenic	*CHMP4B, RALY-AS1*	74812; 63309	20q11.22	
HPV16:3327,chr6:7328094	51/0/131	E2	Chr6	Intronic	*CAGE1*	.	6p24.3	
HPV16:2623,chr6:8814271	37/64/46	E1	Chr6	Intergenic	*LOC100506207, TFAP2A*	28593; 1582645	6p24.3	
HPV16:5534,chrX:20464412	132/168/43	L2	ChrX	Intergenic	*RPS6KA3, CNKSR2*	179662; 928004	Xp22.12	
HPV16:4680,chr6:7326060	43/6/22	L2	Chr6	Downstream	*CAGE1*	.	6p24.3	
HPV16:3163,chrX:20462930	50/0/21	E2	ChrX	Intergenic	*RPS6KA3, CNKSR2*	178180; 929486	Xp22.12	
HPV16:7619,chr2:99404946	5/1/0	LCR	Chr2	Intergenic	*LOC101927070, KIAA1211L*	16585; 5363	2q11.2	S
HPV16:3873,chr2:197526852	5/4/0	E5	Chr2	Intronic	*CCDC150*	.	2q33.1	
HPV16:2891,chr6:12645768	13/25/12	E2	Chr6	Intergenic	*EDN1, PHACTR1*	348341; 71120	6p24.1	
HPV16:3182,chr20:32516399	23/0/8	E2	Chr20	Intergenic	*CHMP4B, RALY-AS1*	74226; 63895	20q11.22	
HPV16:850,chr20:32515476	13/2/8	E7	Chr20	Intergenic	*CHMP4B, RALY-AS1*	73303; 64818	20q11.22	
HPV16:4453,chr20:32472390	25/19/4	L2	Chr20	Intergenic	*CHMP4B, RALY-AS1*	30217; 107904	20q11.22	
HPV16:5744,chr11:103893587	37/62/2	L1	Chr11	Intronic	*PDGFD*	.	11q22.3	
HPV16:7519,chr2:197519914	19/56/0	LCR	Chr2	Intronic	*CCDC150*	.	2q33.1	S
HPV16:4489,chr11:31749949	17/32/0	L2	Chr11	Intronic	*ELP4*	.	11p13	S
HPV16:2110,chr2:99396966	17/25/0	E1	Chr2	Intergenic	*LOC101927070, KIAA1211L*	8605; 13343	2q11.2	S
HPV16:322,chr20:32486709	7/21/0	E6	Chr20	Intergenic	*CHMP4B, RALY-AS1*	44536; 93585	20q11.22	S
HPV16:1276,chr18:50054	5/20/14	E1	Chr18	Intergenic	*LOC102723376, ROCK1P1*	34124; 59011	18p11.32	
HPV16:4394,chr12:17224129	3/15/2	L2	Chr12	Intergenic	*SKP1P2, LINC02378*	80567; 510628	12p12.3	
HPV16:4834,chrX:20461879	41/12/0	L2	ChrX	Intergenic	*RPS6KA3, CNKSR2*	177129; 930537	Xp22.12	S
HPV16:1037,chrX:20445300	2/11/0	E1	ChrX	Intergenic	*RPS6KA3, CNKSR2*	160550; 947116	Xp22.12	
HPV16:6618,chr20:32506419	3/9/12	L1	Chr20	Intergenic	*CHMP4B, RALY-AS1*	64246; 73875	20q11.22	
HPV16:2638,chr20:32502300	4/8/0	E1	Chr20	Intergenic	*CHMP4B, RALY-AS1*	60127; 77994	20q11.22	
HPV16:1155,chrX:20447276	27/8/0	E1	ChrX	Intergenic	*RPS6KA3, CNKSR2*	162526; 945140	Xp22.12	S
HPV16:214,chr6:7242229	6/7/0	E6	Chr6	Intronic	*RREB1*	.	6p24.3	
HPV16:4307,chr13:74240744	2/6/0	L2	Chr13	Intergenic	*LINC00392, KLF12*	78728; 19405	13q22.1	
HPV16:883,chr20:32487936	4/5/5	E1	Chr20	Intergenic	*CHMP4B, RALY-AS1*	45763; 92358	20q11.22	
HPV16:4250,chr2:197550764	2/4/0	L2	Chr2	Intronic	*CCDC150*	.	2q33.1	
HPV16:7362chr18:12717	8/3/0	LCR	Chr18	ncRNA_intronic	*LOC102723376*	.	18p11.32	
HPV16:4405,chr2:99438640	1/3/2	L2	Chr2	Exonic	*KIAA1211L*	.	2q11.2	
HPV16:462,chr20:30942164	8/2/0	E6	Chr20	Intergenic	*KIF3B, ASXL1*	19353; 3983	20q11.21	S
HPV16:6558,chr2:99431140	3/1/0	L1	Chr2	Intronic	*KIAA1211L*	.	2q11.2	
HPV16:7139,chr20:32478733	169/0/0	L1	Chr20	Intergenic	*CHMP4B, RALY-AS1*	36560; 101561	20q11.22	S
HPV16:4276,chr20:32502143	51/0/0	L2	Chr20	Intergenic	*CHMP4B, RALY-AS1*	59970; 78151	20q11.22	S
HPV16:4029,chr11:103891933	21/0/0	E5	Chr11	Intronic	*PDGFD*	.	11q22.3	S
HPV16:3312,chr6:17081322	8/0/0	E2	Chr6	Intergenic	*LOC101928433, STMND1*	314208; 21167	6p22.3	S
HPV16:3295,chr11:31761709	6/0/0	E2	Chr11	Intronic	*ELP4*	.	11p13	S
HPV16:5311,chr1:455824	6/0/0	L2	Chr1	Intergenic	*OR4F16, LOC101928626*	87227; 106936	1p36.33	
HPV16:4027,chr11:103891932	4/0/0	E5	Chr11	Intronic	*PDGFD*	.	11q22.3	
HPV16:6681,chr10:80475	4/0/0	L1	Chr10	Intergenic	*NONE, TUBB8*	NONE; 12353	10p15.3	
HPV16:1570,chrX:20385416	4/0/0	E1	ChrX	Intergenic	*RPS6KA3, CNKSR2*	100666; 1007000	Xp22.12	
HPV16:4508,chr1:143415731	3/0/0	L2	Chr1	Intergenic	*LOC102723769, MIR6077*	213492; 257190	1q21.1	
HPV16:6840,chr6:171021105	3/0/0	L1	Chr6	Intergenic	*PDCD2, NONE*	127325; NONE	6q27	
HPV16:6840,chr1:547221	3/0/0	L1	Chr1	Intergenic	*OR4F16, LOC101928626*	178624; 15539	1p36.33	
HPV16:6840,chr1:547739	3/0/0	L1	Chr1	Intergenic	*OR4F16, LOC101928626*	179142; 15021	1p36.33	
HPV16:6305,chr2:197525082	3/3/0	L1	Chr2	Intronic	*CCDC150*	.	2q33.1	
HPV16:4730,chr1:10270	3/0/0	L2	Chr1	Intergenic	*NONE, DDX11L1*	NONE; 1604	1p36.33	
HPV16:6929,chr2:99443220	2/0/0	L1	Chr2	Intronic	*KIAA1211L*	.	2q11.2	
HPV16:1469,chr3:189605104	2/0/0	E1	Chr3	Intronic	*TP63*	.	3q28	
HPV16:4730,chrX:155259784	2/0/0	L2	ChrX	Intergenic	*DDX11L16, NONE*	1936; NONE	Xq28	
HPV16:4732,chr2:114360505	2/0/0	L2	Chr2	ncRNA_intronic	*DDX11L2*	.	2q13	
HPV16:2621,chr20:3560423	2/0/0	E1	Chr20	Intronic	*ATRN*	.	20p13	
HPV16:4732,chr18:10186	2/0/0	L2	Chr18	Intergenic	NONE, *LOC102723376*	NONE; 1889	18p11.32	
HPV16:3324,chr6:7328088	2/0/0	E2	Chr6	Intronic	*CAGE1*	.	6p24.3	
HPV16:6840,chr1:547835	2/0/0	L1	Chr1	Intergenic	*OR4F16, LOC101928626*	179238; 14925	1p36.33	
HPV16:4793,chr12:80918599	2/0/0	L2	Chr12	Intronic	*PTPRQ*	.	12q21.31	
HPV16:3340,chr15:40890192	2/0/0	E2/E4	Chr15	Intronic	*KNL1*	.	15q15.1	
HPV16:4732,chr1:10164	2/0/0	L2	Chr1	Intergenic	*NONE, DDX11L1*	NONE; 1710	1p36.33	
HPV16:6899,chr2:72209094	2/0/0	L1	Chr2	Intergenic	*DYSF, CYP26B1*	295201; 147273	2p13.2	
HPV16:3340,chr20:35719229	0/35/0	E2/E4	Chr20	Intronic	*RBL1*	.	20q11.23	
HPV16:3340,chr12:81091809	0/96/0	E2/E4	Chr12	Intergenic	*PTPRQ, MYF6*	17841; 9599	12q21.31	
HPV16:1355,chr2:99429402	0/6/0	E1	Chr2	Intronic	*KIAA1211L*	.	2q11.2	
HPV16:4782,chr2:99435835	0/6/0	L2	Chr2	Intronic	*KIAA1211L*	.	2q11.2	
HPV16:1030,chr9:123681030	0/4/0	E1	Chr9	Intronic	*TRAF1*	.	9q33.2	
HPV16:3378,chr17:69506855	0/4/0	E2/E4	Chr17	Intergenic	*CASC17, LINC02095*	308535; 511137	17q24.3	
HPV16:1515,chr21:39554078	0/3/0	E1	Chr21	Intergenic	*DSCR8, DSCR10*	25473; 24172	21q22.13	
HPV16:1916,chr16:79637361	0/3/0	E1	Chr16	Intergenic	*MAF, MAFTRR*	2739; 117848	16q23.2	
HPV16:2117,chr1:240419064	0/3/0	E1	Chr1	Intronic	*FMN2*	.	1q43	
HPV16:3050,chr17:65126150	0/3/0	E2	Chr17	Intronic	*HELZ*	.	17q24.2	
HPV16:3337,chr14:46921971	0/3/0	E2/E4	Chr14	ncRNA_intronic	*LINC00871*	.	14q21.2	
HPV16:4399,chr2:127164444	0/3/0	L2	Chr2	Intergenic	*LINC01941, GYPC*	288882; 249067	2q14.3	
HPV16:4409,chr3:45985168	0/3/0	L2	Chr3	Intronic	*CXCR6, FYCO1*	.	3p21.31	
HPV:16:5685,chr3:56156825	0/3/0	L1	Chr3	Intronic	*ERC2*	.	3p14.3	
HPV16:7086,chr2:99457820	0/3/0	L1	Chr2	Intronic	*KIAA1211L*	.	2q11.2	
HPV16:2724,chr20:30948621	0/0/8	E1	Chr20	Intronic	*ASXL1*		20q11.21	
HPV16:3883,chr3:156990726	0/0/4	E5	Chr3	Intronic	*VEPH1*		3q25.31	
HPV16:3344,chr6:7327990	0/89/43	E2	Chr6	Intronic	*CAGE1*	.	6p24.3	
HPV16:1381,chr11:31187618	0/3/0	E1	Chr11	Intronic	*DCDC1*	.	11p13	
HPV16:1891,chr20:60384079	0/3/0	E1	Chr20	Intronic	*CDH4*	.	20q13.33	
HPV16:2075,chr12:39215471	0/3/0	E1	Chr12	Intronic	*CPNE8*	.	12q12	
HPV16:2237,chr4:28262779	0/3/0	E1	Chr4	Intergenic	*LINC02261,MIR4275*	978932;558425	4p15.1	
HPV16:2716,chr13:74250432	1/3/0	E1	Chr13	Intergenic	*LINC00392,KLF12*	88416;9717	13q22.1	
HPV16:3340,chr12:81091909	0/58/0	E2/E4	Chr12	Intergenic	*PTPRQ,MYF6*	17941;9499	12q21.31	
HPV16:2724,chr20:30948899	0/11/0	E1	Chr20	Intronic	*ASXL1*	.	20q11.21	
HPV16:3340,chr1:212166417	0/27/0	E2/E4	Chr1	Intronic	*INTS7*	.	1q32.3	

*Breakpoint: each breakpoint was named by combined breakpoint site of HPV genome, chromosome and chromosome location of human breakpoint; Reads Number: N (Nanopore), I (Illumina), Liu (Liu et al.), Nanopore reads number cut-off value is 2, and Illumina reads number cut-off value is 3. There is no cut-off value for integration sites which overlapped by different sequencing platforms. Lane of GeneDetail_refGene listed the distance of integration sites with the nearby genes. S:Sanger Verified.

With the comparison of the three integration datasets, Illumina, Nanopore, and Liu et al., we found that although overlapping results were found by all of them, there were still unique breakpoints identified by each dataset. Our Illumina data contained 22 sites that were not identified by nanopore and previous studies. Although nanopore results provided good coverage of published data, it still missed some sites by our data analysis, possibly caused by the differences in the library construction step. The capture efficiency of the probes might be different when using different lengths of DNA fragments; therefore, some integration sites may not be seen in the other platform. We also noticed that most of the unmatched integration sites had relatively low read numbers covered. Among the 22 unique Illumina breakpoints, There were 13 breakpoints with only three reads being identified. We also compared all of the breakpoints identified between the two platforms; those with only one read had few overlaps. Therefore, we hypothesize that the capture efficiency of probes with low abundant reads varies more compared with high abundant reads. Since it is difficult to verify the low read counts by Sanger sequencing or other methods, we are not sure if the 280 integration sites we found with only one read are real integration sites or not. Since each data analysis method has its limitations based on the mechanism of the method, our conclusion was drawn based on the methods we chose for this study. There might be false positive or false negative integration sites existing in the list. However, due to the technical difficulty of measuring the exact sensitivity and specificity of the method in detecting virus integration sites, the overlapped integration sites in two different data sets and the verified integration sites by Sanger sequencing provided more valuable information for our conclusion. The other integration sites we identified and summarized in the list provided more reference values.

Our data indicated that most of the integration sites exist in the intergenic region of the human genome, concordant with previous studies (Bodelon et al., 2016; Pinatti et al., 2017; Brant et al., 2018; Koneva et al., 2018; Li et al., 2019). Since human genome was identified with large portion (∼60%) of intergenic region, our results identified about 40% integration sites located in intergenic region of human genome, which showed no significant difference. Therefore, it could be concluded that the way that integration sites distributed in human genome was affected by the nature of genome functional structure. We hypothesized that another underlying mechanism might be that the integration of HPV genome to human non-exon region of genes wouldn’t cause significant phenomenon of losing the function of the genes, which therefore could keep the host cell for living, instead of dying instantly. We also found that integration happened in non-coding RNA (ncRNA), which plays many important functions, for example, long ncRNA interacts with p53 protein (Liu Y. et al., 2019). Insertion of ncRNA might cause more serious function interruption; therefore, the incidence is not very high. Some genes have been found to be hot spot genes, in which HPV has the tendency to insert, for example tumor protein 63 (*TP63*), which plays very important roles in carcinogenesis (Ojesina et al., 2014). Akagi et al. (2014) identified integration happened in *TP63*. In this study, we identified hotspots in the intergenic region of two genes *CHMP4B* and *RALY-AS1*. The integration cluster tendency was very strong, with 10 integration sites located in 76 unique integration sites identified, as well as in several other chromosome regions. Why does the virus integrate into several specific regions and is it random or specific? Further studies are needed to answer these questions.

The significantly increased gene expression of *HMGA2* and *TP63* has been reported in neoplastic samples where HPV is integrated into their introns, whereas *LRP1B* is under expression with HPV integration in their flanking region (Michael et al., 2014; Hu et al., 2015). Therefore, a combination of *HMGA2, LRP1B*, and *TP63* as potential biomarkers may be useful for screening during triage of HPV-positive patients, particularly for detecting CIN2+ (Jiang et al., 2019).

In recent integration sites analysis, most of reported genes affected by viral genome integration are related to cellular repair pathways, tumor suppression, growth, and cell proliferation, and in some cases, code for transcription factors (Oyervides-Muñoz et al., 2018). Several studies aiming to discover viral integration sites in genome of host cells have demonstrated frequent in the *MYC, TMEM49*, and *FANCC* genes (Zhang et al., 2016). In another report, *POU5F1B*, *FHIT*, *KLF12*, *KLF5*, *LRP1B*, and *LEPRL1* were found to be recurrent sites for integration (Hu et al., 2015). Interestingly, *FHIT* gene has been associated translocation in cancer while the *LEPREL1* gene has also been associated with breast cancer development. In our study, we found viral integration in the *PDGFD, ELP4*, and *ATRN* genes, with roles in the regulation of cell proliferation, transcription, and DNA-templated and inflammatory response, which were not reported by other neoplastic studies.

We believe that nanopore has obvious advantages compared with Illumina sequencing, as reported previously (Xiao et al., 2018; Liu Q. et al., 2019). In this study, we generated about 150 M data by nanopore and obtained better results than Illumina sequencer, which generated 1.6 G data and was ten times greater than nanopore. Besides the sequencing data amount, the instant data analysis capability would make the application more efficient and prompter, which is important for clinical usage. These will bring huge potential for nanopore application in multiple areas of clinical diagnosis, especially pathogen detection. We think our method developed for HPV and its integration site detection will become an important tool in the research or clinical related application field in the future.

There was only few newly published paper using nanopore platform to identify HPV integration sites (Quan et al., 2019; Van Arsdale et al., 2019). The first study revealed that using nanopore sequencing could simultaneously detect HPV infection and microbiota composition promptly and accurately. The other showed that long-range DNA sequencing utilizing an Oxford Nanopore MinION flowcell yielded 3.56 × haploid genome coverage including three reads encompassing the HPV70 DNA insertion in *BCL11B*. The latter also provides evidence of feasibility in using nanopore in clinical applications of virus integration detection, which confirmed our conclusion in another way.

## Conclusion

By using nanopore sequencing technology, we successfully enriched the virus DNA sequence and virus integrated human genome sequence with novel discoveries compared with previous published. These data strongly suggest that nanopore can be used for both prompt virus detection and infection status discovery with comparable accuracy of Illumina but with far more less required sequencing data, and without the need for error correction.

## Data Availability Statement

The datasets generated for this study can be found in the Genbank database repository, PRJNA591355, https://www.ncbi.nlm.nih.gov/sra/PRJNA591355.

## Ethics Statement

The studies involving human participants were reviewed and approved by the Institutional Review Board of the Peking University School of Oncology, China. The patients/participants provided their written informed consent to participate in this study.

## Author Contributions

GT and YL acquired the funding for this study. BM and GT conceptualized the study. YL and JjL obtained the samples, patient consent letters, and ethical approval letter for this study. WY, JL, and WW conducted the experiments and collected the data. BM, RD, and JdL analyzed most of the results. BM and WY wrote the initial draft of the manuscript. BM, YL, JY, WW, and GT revised the manuscript. All authors discussed the results and reviewed the manuscript.

## Conflict of Interest

WY, RD, JL, JdL, JY, WW, BM, and GT was employed by company Geneis (Beijing) Co., Ltd, China. The remaining authors declare that the research was conducted in the absence of any commercial or financial relationships that could be construed as a potential conflict of interest.

## Footnotes

1 http://hannonlab.cshl.edu/fastx_toolkit/

2 http://www.openbioinformatics.org/annovar/annovar_download.html
