# Supplementary material for: Accurate Detection of HPV Integration Sites in Cervical Cancer Samples Using the Nanopore MinION Sequencer Without Error Correction
Source: Front Genet. 2020 Jun 26;11:660. doi: 10.3389/fgene.2020.00660 (PMC7344299; doi:10.3389/fgene.2020.00660)

**Figure S1.** Sequence depth coverage diagram of Illumina and nanopore results on HPV16 genome.

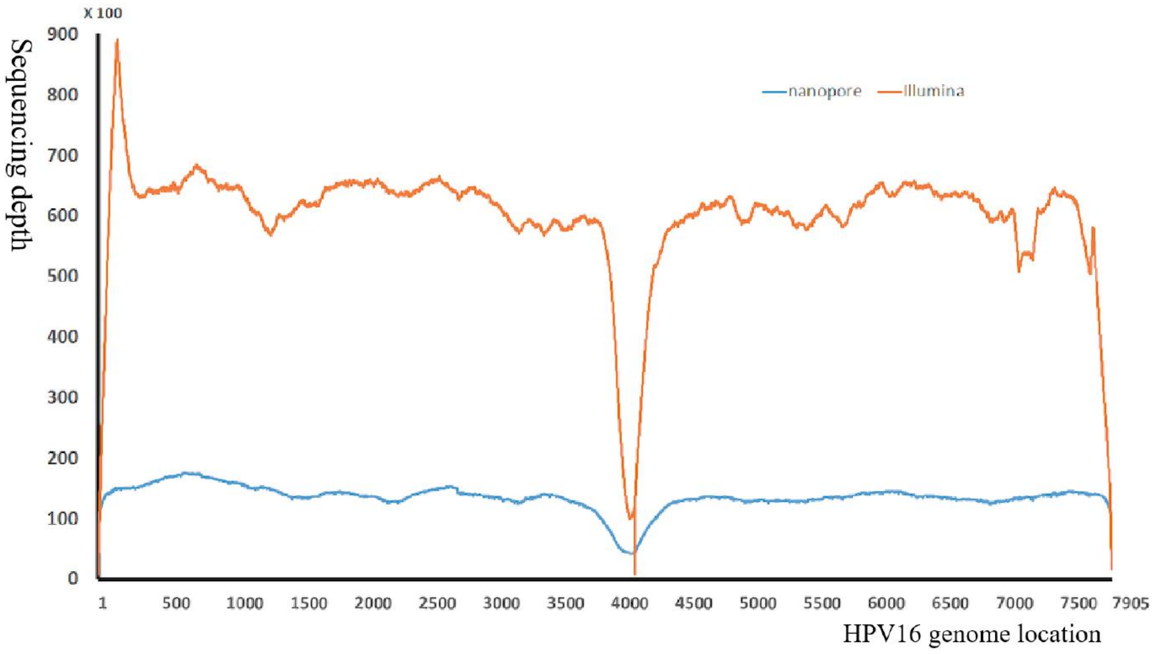


**Figure S2**. Sanger sequencing chrome image of all 13 verified integration sites.

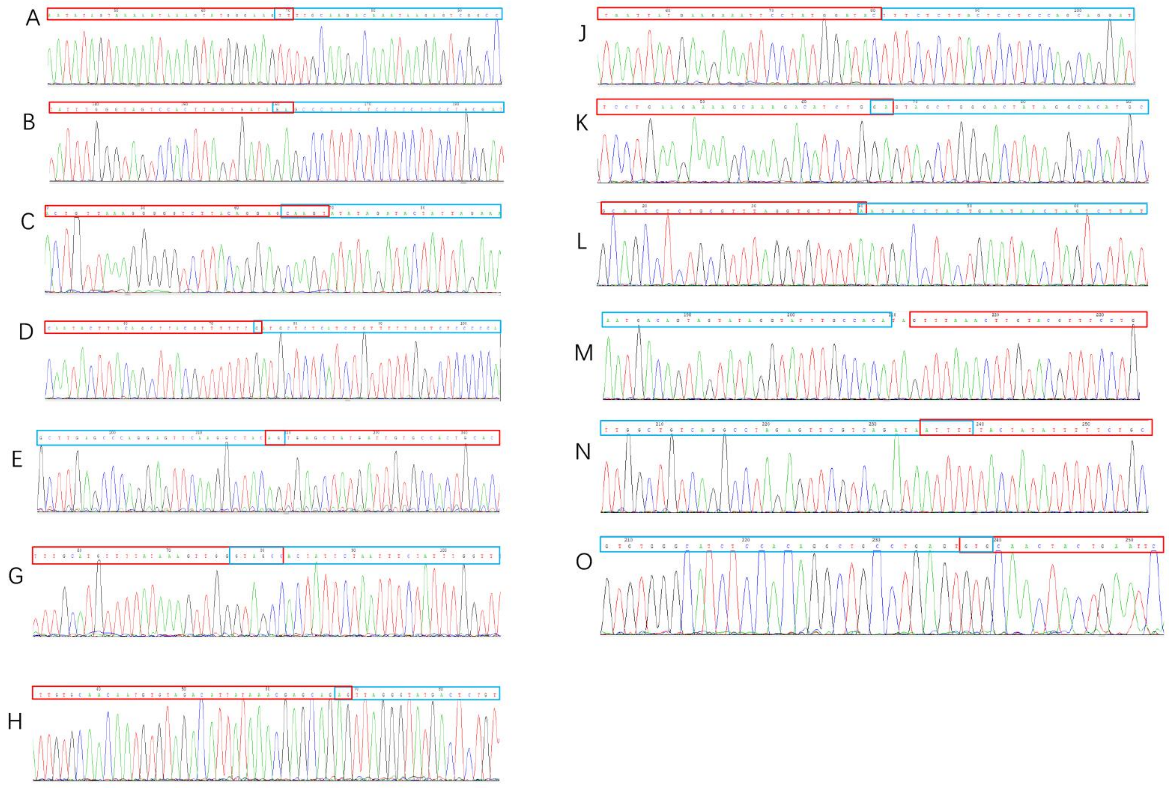

**Figure S3**. Function classification of the unique integrated genes.

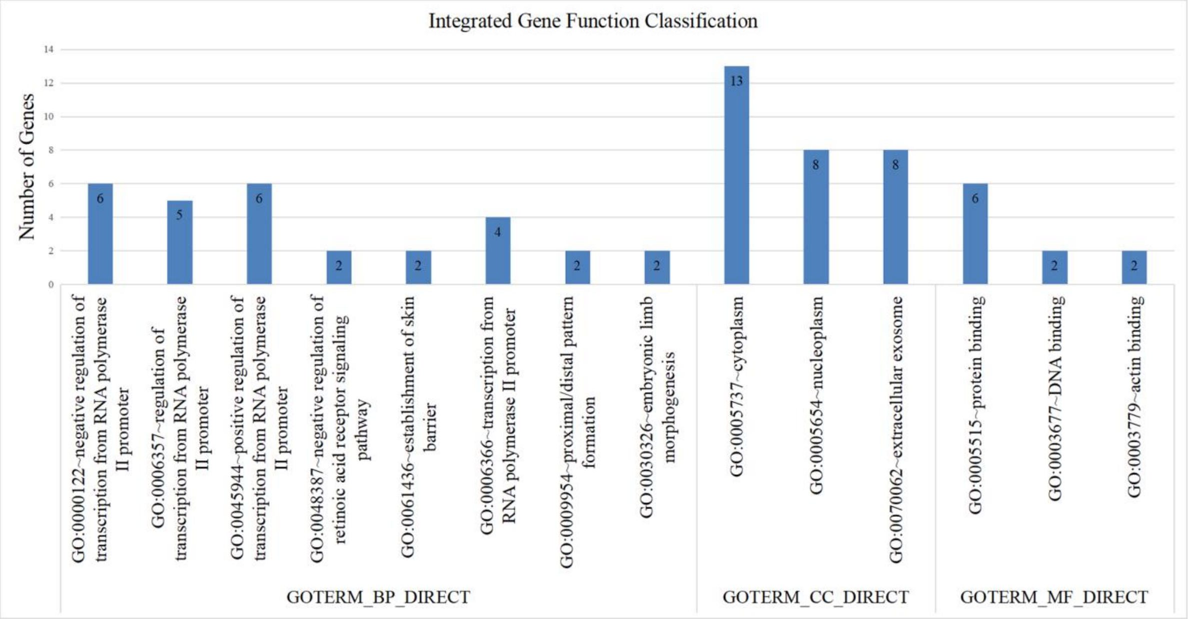

Supplement: Supplementary file 2 [file Presentation_1.pdf]
